# Fedratinib-Associated Uveitis Effectively Treated With Sub-Tenon's Triamcinolone Acetonide Injection: A Case Report

**DOI:** 10.7759/cureus.81184

**Published:** 2025-03-25

**Authors:** Akira Saitoh, Ayumi K Saitoh, Erina Daizumoto, Yukiko Nagahara

**Affiliations:** 1 Ophthalmology, Takamatsu Red Cross hospital, Takamatsu, JPN; 2 Ophthalmology, Nishihara Eye Clinic, Takamatsu, JPN; 3 Ophthalmology, Kagawa University, Takamatsu, JPN

**Keywords:** drug-induced uveitis, fedratinib, janus kinase 2 inhibitor, myelofibrosis, triamcinolone acetonide injection

## Abstract

This report describes the case of uveitis in a 74-year-old woman with myelofibrosis who was treated with fedratinib. Both her eyes had hyperemia, anterior chamber cells, keratic precipitates, and vitritis. Based on her clinical history, negative clinical examination findings (except for diabetes), and fedratinib treatment, she was diagnosed with fedratinib-induced uveitis. Subsequently, the patient received sub-Tenon's triamcinolone acetonide (STA) injection in both eyes. The next day, her subjective symptoms were alleviated. Moreover, her objective symptoms were deemed to have improved after the examination performed after a few days. STA injection was extremely effective for treating fedratinib-induced uveitis.

## Introduction

Uveitis is defined as inflammation of the uvea (iris, ciliary body, and choroid). However, the retina, the aqueous humor in the anterior chamber, and the vitreous humor are also often affected. Approximately half of the cases are idiopathic, identifiable causes include trauma, infections, and systemic diseases, many of which are autoimmune [1]. 

Myelofibrosis (MF) is a hematological condition characterized by uncontrolled bone marrow proliferation often attributed to acquired driver mutation-mediated constitutive activation of the Janus kinase 2 (JAK2)-signal transducer and the activator of the transcription signaling pathway [2]. 

After the approval of ruxolitinib in 2011, the use of fedratinib for the treatment of adult patients with adult intermediate-2 or high-risk primary and secondary MF was authorized by the United States Food and Drug Administration (FDA) in 2019 [2-4]. As per a previous study, fedratinib has various side effects [5]. However, in the field of ophthalmology, side effects have been rarely reported [3,4]. 

To the best of our knowledge, there have been reports of only one case of fedratinib-associated uveitis [4] and one case of fedratinib-induced orbital inflammation [3]. In the former case of uveitis, the patient received steroid eye drops and gradually recovered after several months of treatment [4]. In the latter case, the patient recovered after the discontinuation of fedratinib [3].

Herein, we describe a case of uveitis in a 74-year-old woman with MF who was treated with fedratinib after ruxolitinib therapy. In this case, treatment with sub-Tenon's triamcinolone acetonide (STA) injection in both eyes was effective. 

## Case presentation

A 74-year-old woman presented to the Nishihara Eye Clinic with a six-day history of decreased visual acuity, hyperemia, floating dots, and right eye pain. There were no symptoms suggesting uveitis. Past medical history included MF and diabetes but no ophthalmic history other than the incipient cataract. Four years prior to the presentation, the patient had been diagnosed with MF and treated with ruxolitinib at the Department of Hematology, Takamatsu Red Cross Hospital. Two years ago, the treatment at the Department of Hematology, Takamatsu Red Cross Hospital, was switched from ruxolitinib to fedratinib.

**Figure 1:**
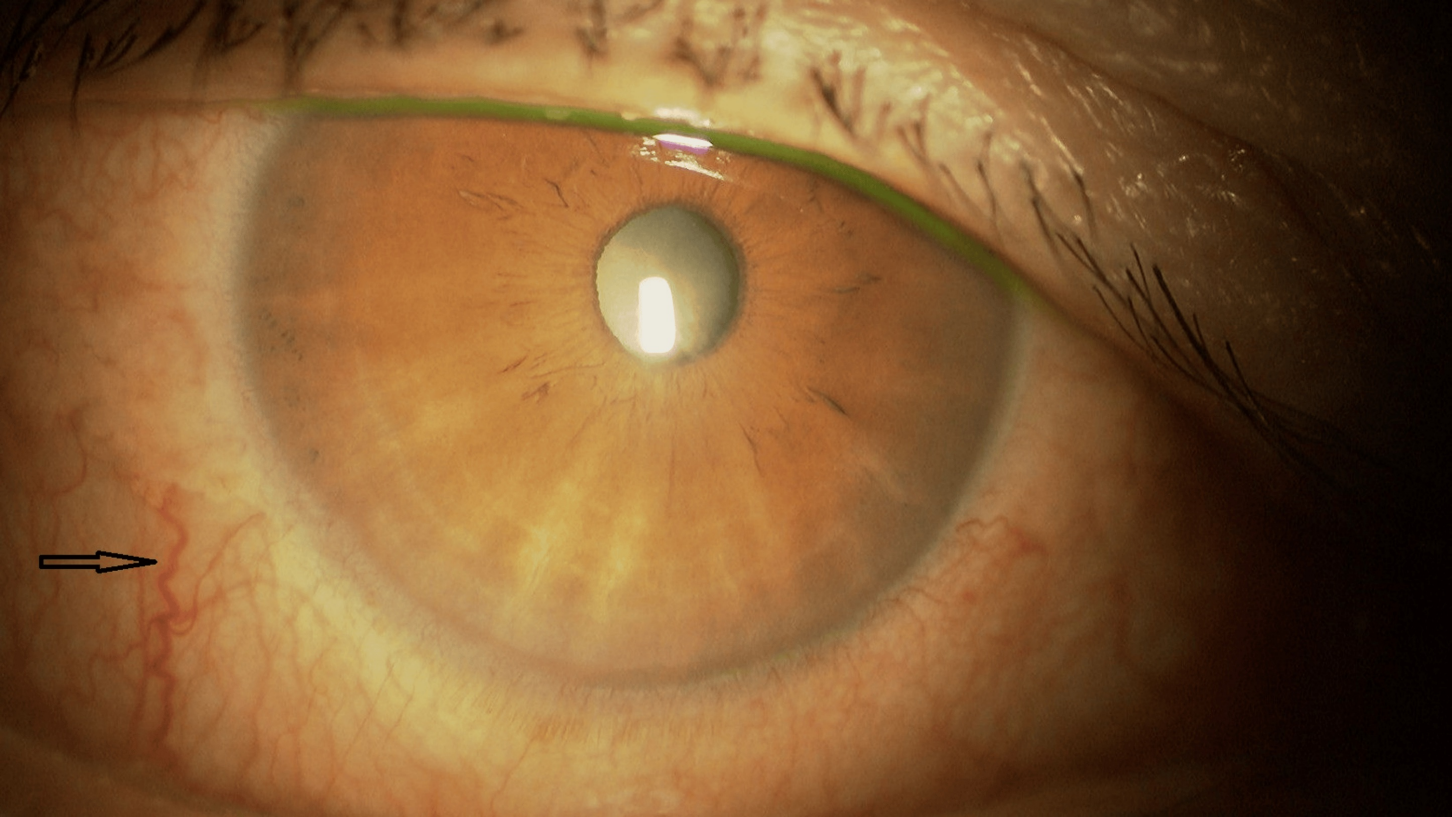
Anterior segment image of the right eye showing conjunctival hyperemia and incipient cataract

**Figure 2:**
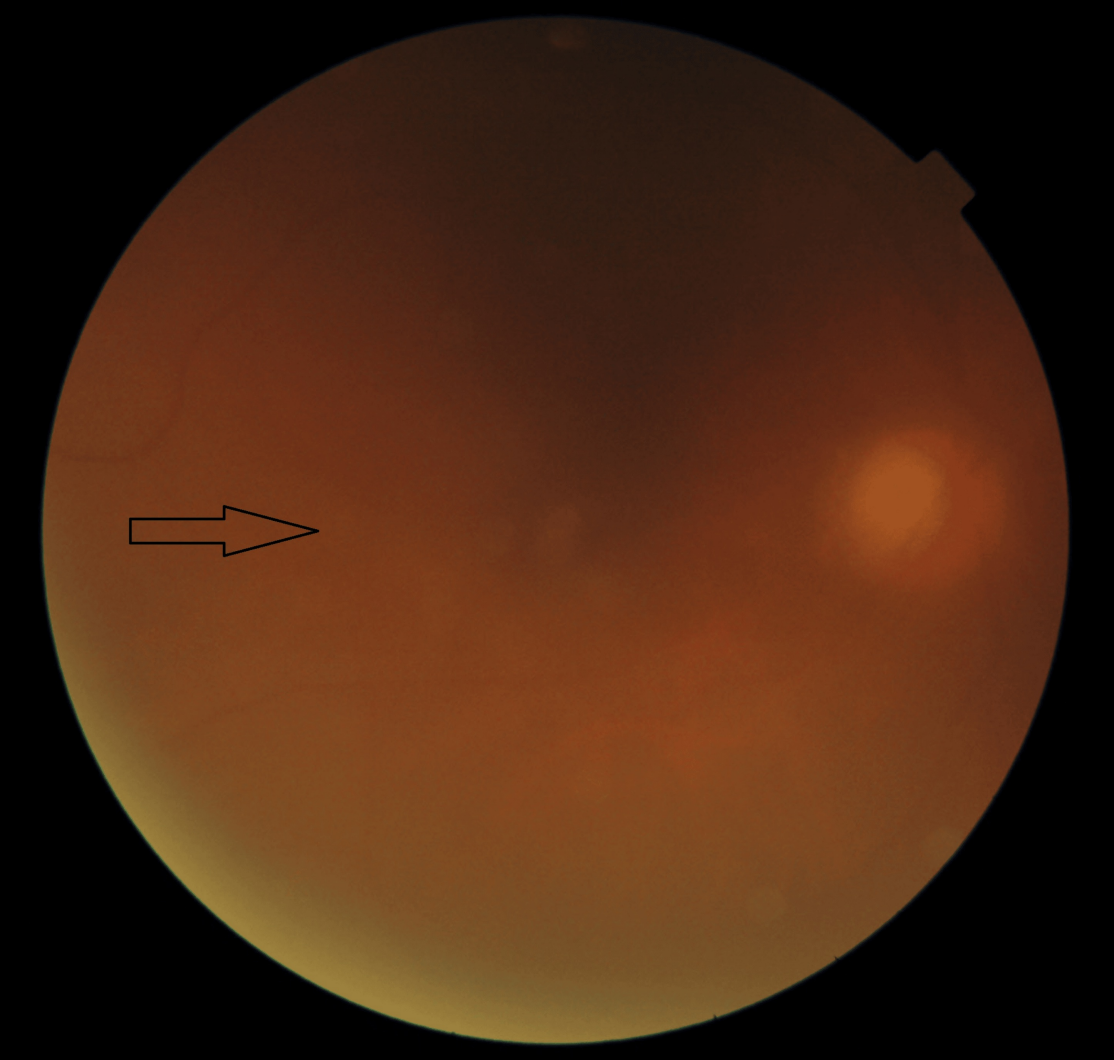
Fundus image of the right eye showing diffuse vitreous opacity

**Figure 3:**
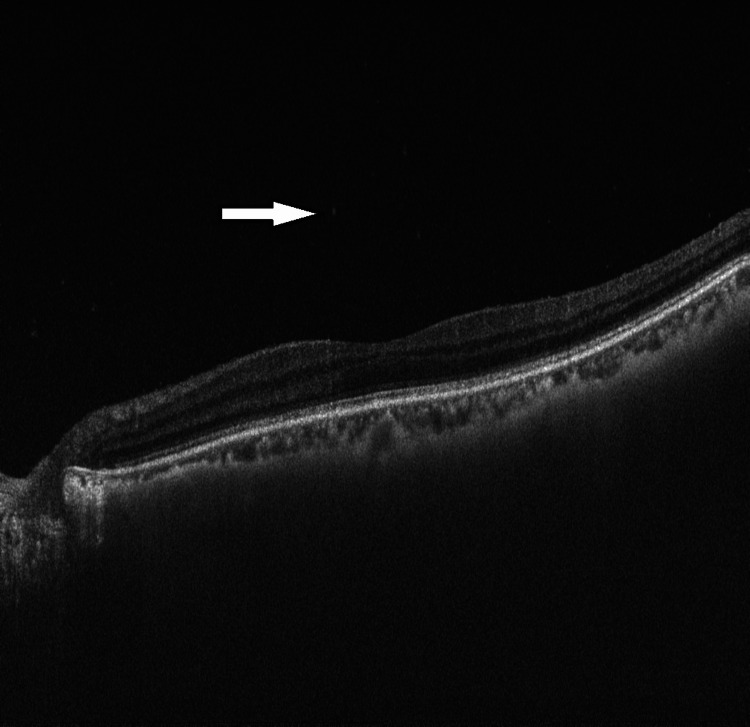
Ocular coherent tomography of the right eye showing vitreous opacity with vitreous cells. However, there was no evidence of macular edema.

At presentation, the best corrected visual acuity (BCVA) values were 0.4 in the right eye and 0.8 in the left eye. The intraocular pressure (IOP) values were 16 mmHg in the right eye and 15 mmHg in the left eye. A slit-lamp biomicroscopy examination revealed incipient cataract and conjunctival hyperemia in the right eye with anterior chamber cells, and fine keratic precipitates were present; posterior synechia was not observed. This is illustrated in Figure 1. Fundus examination revealed diffuse vitreous, as can be seen in Figure 2. Ocular coherent tomography (OCT) scan indicated vitreous opacity with vitreous cells. However, there was no evidence of macular edema. These findings are seen in Figure 3. Treatment with topical betamethasone 0.1 %, and levofloxacin 1.5% eye drops was initiated. 

The next day, she was referred to the Department of Ophthalmology, Takamatsu Red Cross Hospital for further examination and treatment.

On presentation at the Department of Ophthalmology, Takamatsu Red Cross Hospital, the BCVA values were 0.7 in the right eye and 1.2 in the left eye. The IOP values were 12 mmHg in the right eye and 14 mmHg in the left eye. A slit-lamp biomicroscopy examination revealed conjunctival hyperemia in the right eye with anterior chamber cells, and the presence of fine keratic precipitates. Fundus examination revealed diffuse vitreous opacity. Retinal hemorrhage, vitreous hemorrhage, retinal exudation, or disc edema were not observed. OCT revealed vitreous opacities with vitreous cells. However, there was no evidence of macular edema. Treatment with topical betamethasone 0.1 %, and levofloxacin 1.5% eye drops was continued.

Oral fedratinib therapy (200 mg a day, owing to JAK-2 pathogenic gene variants) was continued, because the association between fedratinib and uveitis was initially nuclear and fedratinib was effective for splenomegaly.

The patient received the COVID-19 vaccine three times at the time of presentation (the most recent being two years back). Laboratory and radiological tests were performed for the differential diagnosis. Based on the serological tests, the patient tested negative for toxoplasma, herpes simplex virus, varicella-zoster virus, cytomegalovirus, angiotensin-converting enzyme (ACE), human T-lymphotropic virus types 1 and 2: human immunodeficiency virus, *Aspergillus*, tuberculosis, and anti-neutrophilic cytoplasmic autoantibodies: antinuclear antibody: human leukocyte antigen (HLA)-A26, HLA-B27, and HLA-B52. Hemoglobin A1c was 7.2%, and blood glucose level was 163 mg/dL. Table 1 presents the details regarding the serological tests conducted.

**Table 1:** Laboratory findings of the patient HSV: herpes simplex virus; VZV: varicella-zoster virus; CMV: cytomegalovirus; ACE: angiotensin-converting enzyme; HTLV: human T-lymphotropic virus; HIV: human immunodeficiency virus; ANCA: antineutrophilic cytoplasmic autoantibodies (ANCA); PR3: proteinase 3; MPO: myeloperoxidase; ANA: antinuclear antibody; HLA: human leukocyte antigen; HbA1c: glycated hemoglobin

Item	Result	Comment	Reference Range	Unit
Toxoplasma-IgM	Negative			
HSV-IgM	0.15	Negative		
VZV-IgM	0.07	Negative		
CMV-IgM	>0.85	Negative		
ACE	14.2	WNL	7 - 25	U/L
HTLV types 1, 2	0.1	Negative	0.0 -0.9	COI
HIV-Ag, Ab	0.1	Negative	0.0 - 0.9	COI
Aspergillus	0.1	Negative		
T-SPOT-TB (Tuberculosis)	Negative			
PR3-ANCA	>0.6	Negative		IU/mL
MPO-ANCA	>0.6	Negative		IU/mL
ANA Ab	>40	Negative		
HLA-A26	Negative			
HLA-B27	Negative			
HLA-B52	Negative			
Blood glucose level	163	High	73 - 109	mg/dL
HbA1c	7.2	High	4.9 - 6.0	%

Furthermore, the patient’s plain-film chest radiography results were normal. According to the ocular findings, the patient did not present with sarcoidosis, Behçet`s disease, and Vogt-Koyanagi-Harada disease. The patient was receiving treatment for diabetes.

**Figure 4:**
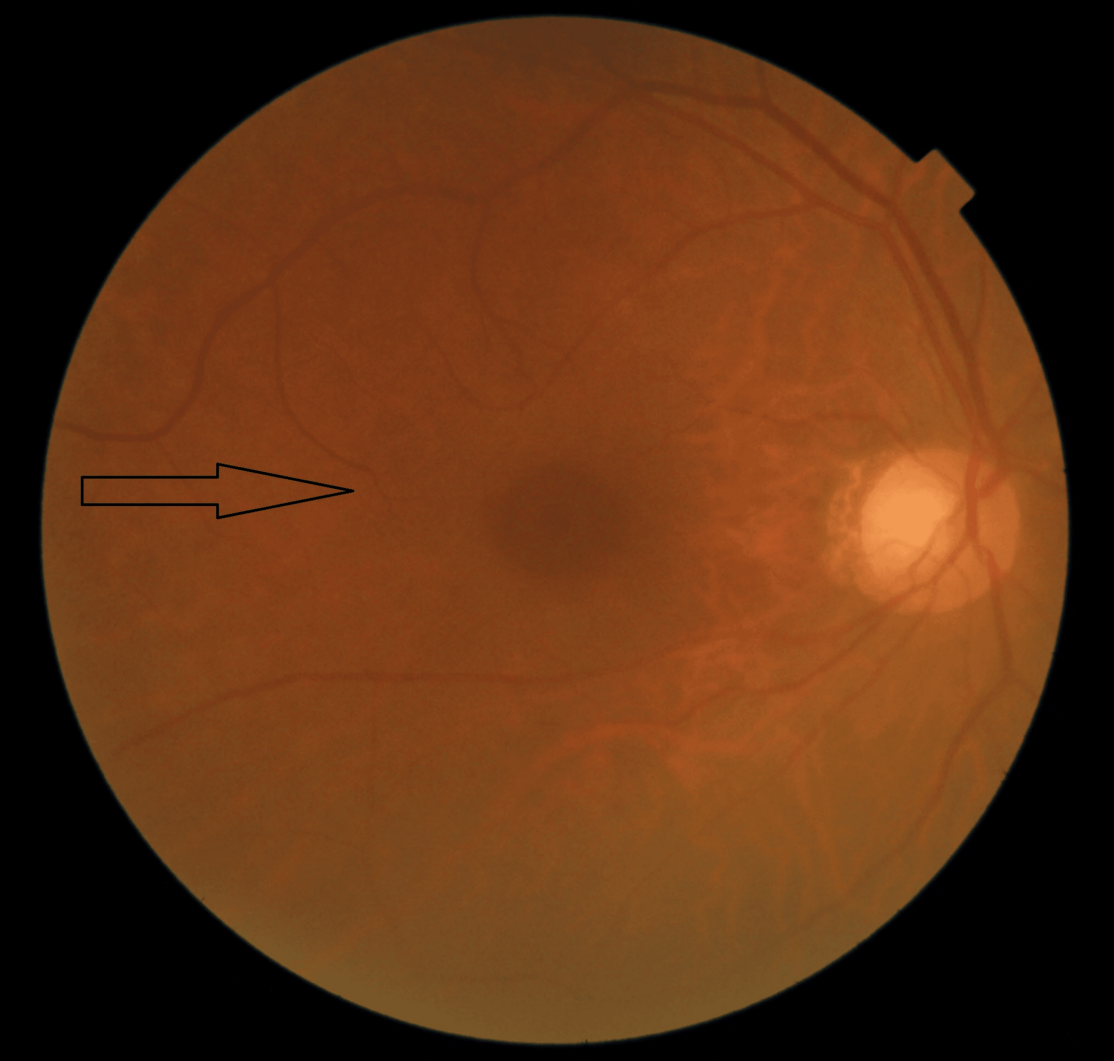
Fundus image of the right eye showing absence of vitreous opacity

**Figure 5:**
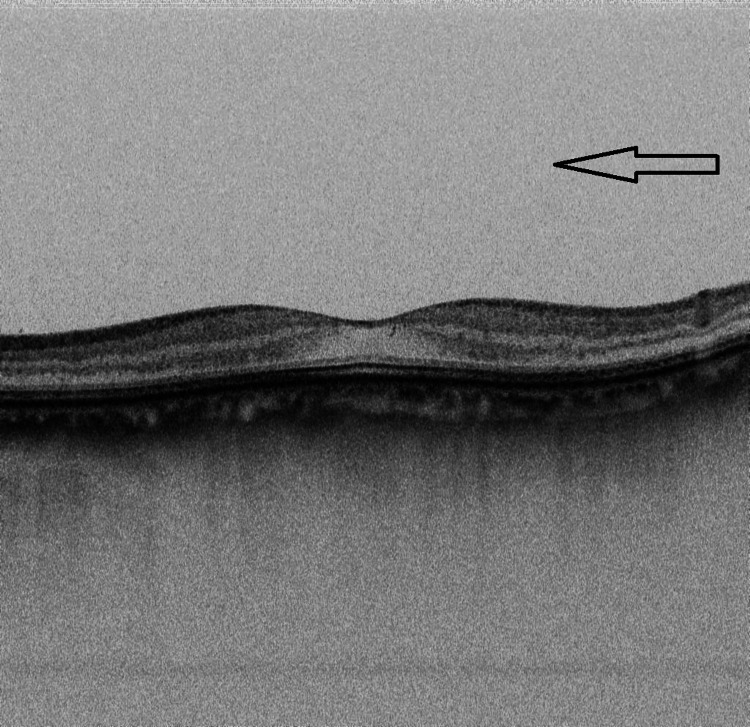
Ocular coherent tomography of the right eye showing absence of vitreous opacity and vitreous cells

Three days after the first visit to the Department of Ophthalmology, Takamatsu Red Cross Hospital, the patient’s ocular pain improved. The BCVA values were 0.8 in the right eye and 1.2 in the left eye. As there was no clinical improvement, STA was performed on the right eye. On the next day, the patient’s subjective symptoms, which included vision, redness, floaters, and eye pain, had disappeared. During the next visit, which was after seven days, her objective symptoms, which included visual acuity, redness, and vitreous opacity observed during ophthalmological examination, also improved. The BCVA values were 1.0 in the right eye and 1.2 in the left eye. The intraocular IOP was 13 mmHg in the right eye and 13 mmHg in the left eye. All of the patient’s clinical findings were alleviated (Figures 4, 5). 

**Figure 6:**
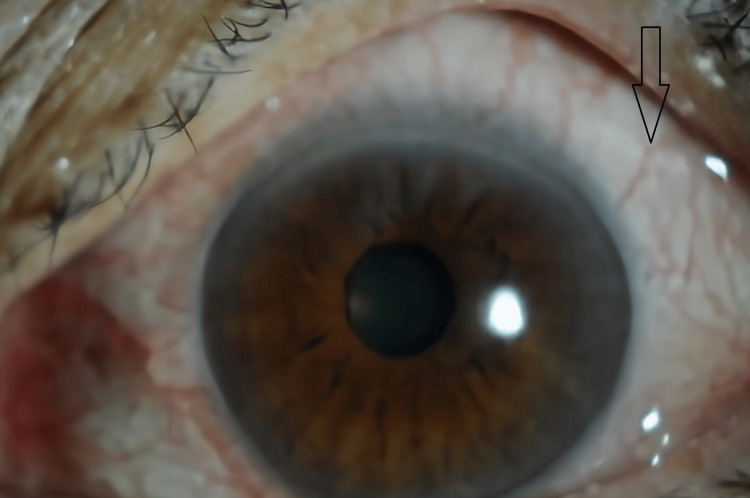
Anterior segment image of the left eye showing conjunctival hyperemia

**Figure 7:**
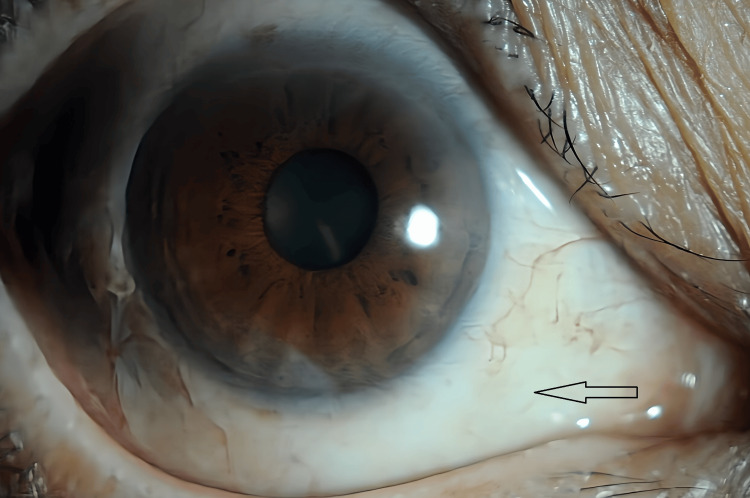
Anterior segment image of the right eye showing absence of hyperemia

Nine days after all clinical symptoms were alleviated, the patient complained of hyperemia and left eye pain. The BCVA values were 1.0 in the right eye and 0.8 in the left eye. The IOP values were 17 mmHg in the right eye and 16 mmHg in the left eye. A slit-lamp biomicroscopy examination revealed conjunctival hyperemia in the left eye with anterior chamber cells, and fine keratic precipitates. Conjunctival hyperemia in the right eye had disappeared. This is seen in Figure 6 for the left eye; the right eye is shown in Figure 7. Fundus examination showed diffuse vitreous opacity. Retinal hemorrhage, vitreous hemorrhage, retinal exudation, and disc edema were not observed. Vitreous opacities with vitreous cells were found on OCT. However, there was no evidence of macular edema.

**Figure 8:**
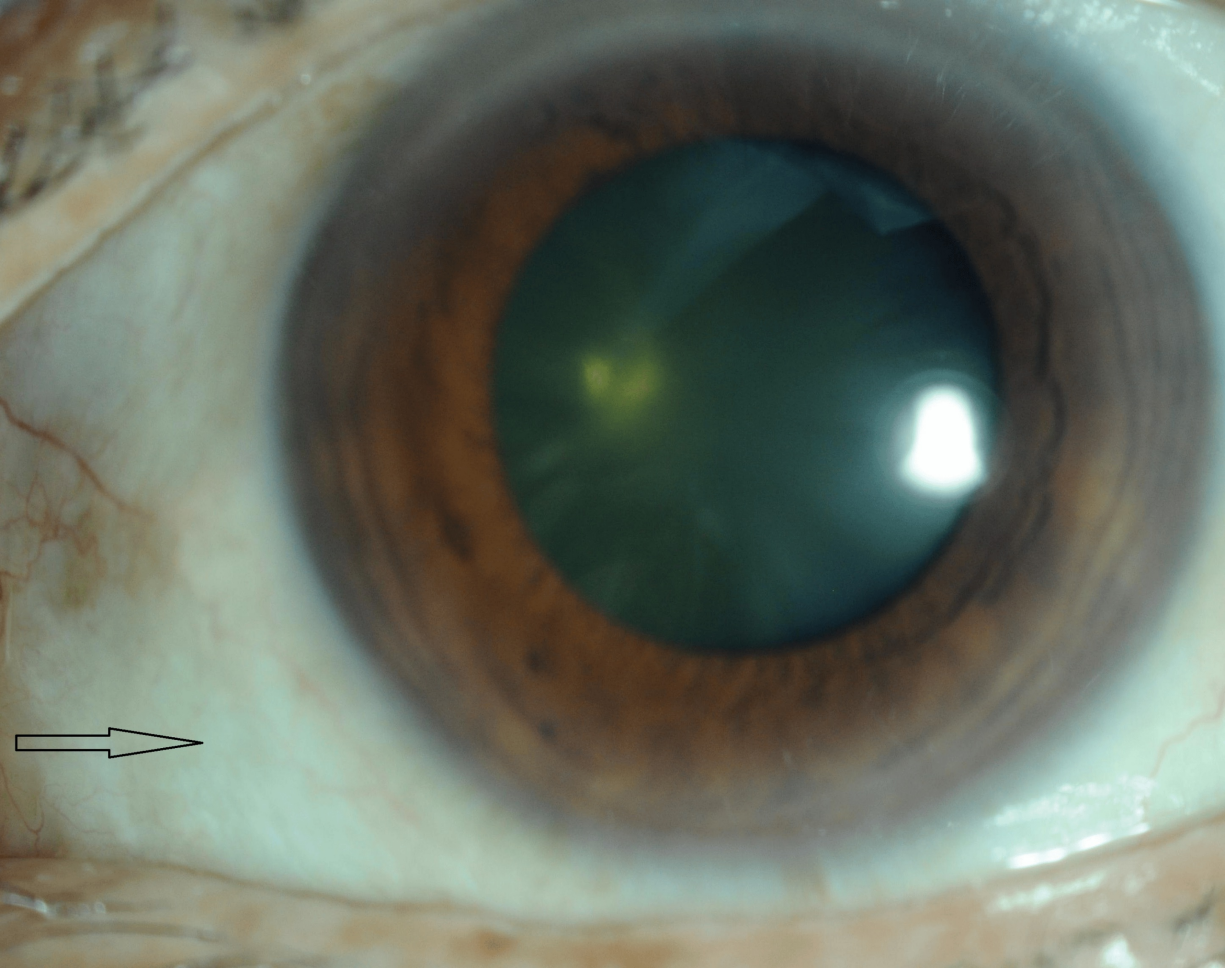
Anterior segment image of the left eye showing disappearance of conjunctival hyperemia

**Figure 9:**
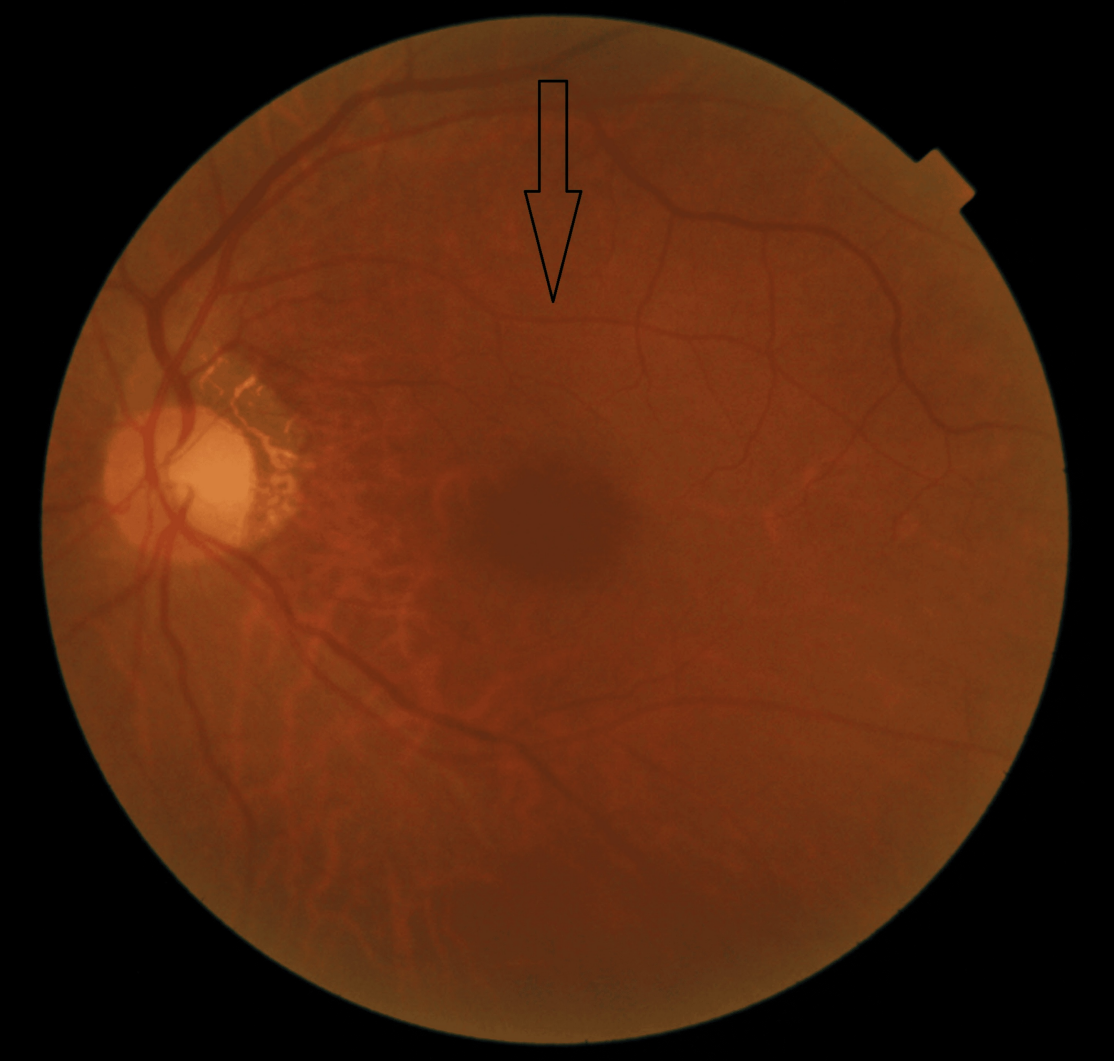
Fundus image of the left eye showing absence of vitreous opacity

**Figure 10:**
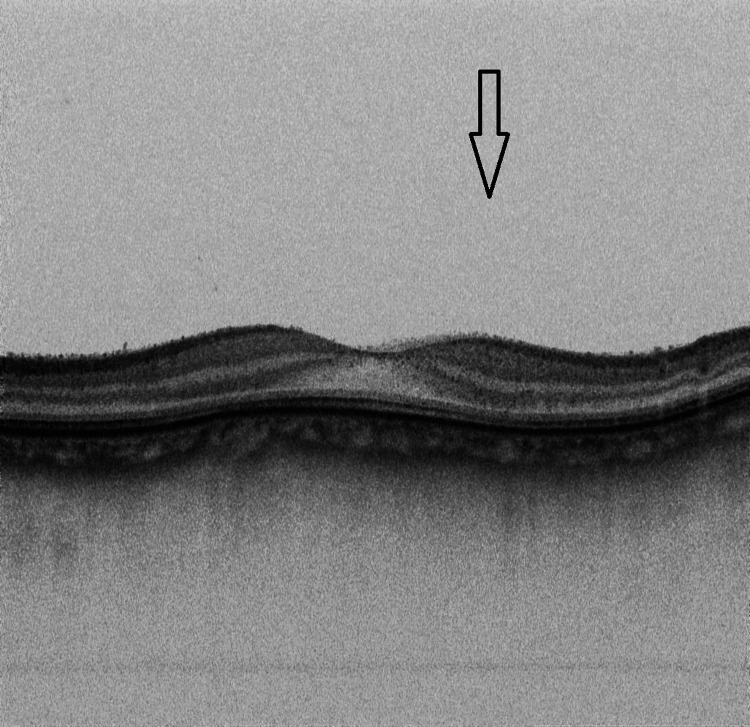
Ocular coherent tomography of the left eye showing absence of vitreous opacity and vitreous cells

Treatment with topical betamethasone 0.1 % and levofloxacin 1.5% eye drops were started on both eyes. STA was conducted on the left eye. On the next day, the patient’s subjective symptoms were alleviated. Moreover, her objective symptoms were completely treated during the follow-up visit after one month. Conjunctival hyperemia of the left eye disappeared. This is seen in Figure 8. Vitreous opacity and vitreous cells of the left eye had disappeared, as can be seen in Figures 9, 10.

The BCVA values were 1.0 in the right eye and 1.0 in the left eye. The IOP values were 12 mmHg in the right eye and 13 mmHg in the left eye. The clinical symptoms continually improved as of five months after the initial diagnosis.

## Discussion

Uveitis is defined as inflammation of the uvea (iris, ciliary body, and choroid). However, the retina, the aqueous humor in the anterior chamber, and vitreous humor are also often affected. Approximately half of the cases are idiopathic; identifiable causes include trauma, infections, and systemic diseases. Symptoms include decreased vision, eye pain, redness, photophobia, and floaters. Uveitis can be clinically identified, but determining its cause usually requires examination. Typically, uveitis is treated with steroid eye drops, injections, or systemic administration [1].

MF is a hematological condition characterized by uncontrolled bone marrow proliferation often attributed to acquired driver mutation-mediated constitutive activation of the JAK2-signal transducer and the activator of the transcription signaling pathway. It can lead to cytopenia, cachexia, extramedullary hematopoiesis-associated splenomegaly (90%), hepatomegaly (50%), and cytokine-driven symptoms including fever, night sweats, itchiness, cachexia, and bone pain [2]. 

Fedratinib and ruxolitinib can help alleviate debilitating symptoms such as splenomegaly, bone pain, and hypermetabolic syndrome [1]. Since the approval of fedratinib (which is the second JAK inhibitor approved for MF after ruxolitinib) in 2019, it has been used on patients with intermediate- or high-risk MF who had failed ruxolitinib treatment [2,3,5,6]. 

In nearly half of all uveitis cases, the causes were not identifiable [7]. The usual etiologies are infections, Behçet's disease, and Vogt-Koyanagi-Harada disease. Diabetic iritis occurs in patients with a hemoglobin A1c level of ≧10, particularly ≧12. Moreover, inflammation is limited in the anterior segments, particularly in the anterior chamber [8]. Coronavirus disease 2019 (COVID-19) vaccine-associated uveitis typically develops within 14 days after receiving the vaccine [9]. However, even though the current patient received the COVID-19 vaccine three times, the most recent was two years earlier and she had no history of COVID-19 infection. Uveitis associated with myelofibrosis involves immune abnormalities, but within the scope of this investigation, no abnormal values were found. Therefore, the above-mentioned causes were excluded.

In approximately 0.5% of cases, uveitis is drug-induced [7]. Naranjo et al. proposed 10 criteria to establish causation between a medication and an adverse reaction [10]. In these criteria, discontinuation and resumption of medication, and dose reduction are important for disease diagnosis. However, in this case, fedratinib therapy (200 mg a day owing to JAK2 pathogenic gene variants) was continued because, initially, the association between fedratinib and uveitis was unclear, and fedratinib was effective for splenomegaly.

The Naranjo scoresheet for quantitatively evaluating the causality between a medication and adverse effects is based on 10 questions, and the scores classify the causality as follows: a definite association (9 or higher), a probable association (5-8), a possible association (1-4), or a doubtful association [10]. This scoresheet does not provide criteria for a conclusive diagnosis. In this case, a score of 5 points was obtained based on the score sheet, which falls into the category of a "probable" association.

Through a systematic and thorough investigation, all potential causes were ruled out. Consequently, a diagnosis of fedratinib-associated uveitis was established. In the field of ophthalmology, there are only a few reports on the side effects of fedratinib [3,4]. A previous study reported ruxolitinib-associated bilateral toxoplasmosis retinitis [11]. In another study in 2024, a 64-year-old man presented with fedratinib-associated uveitis and was treated with topical steroid eye drops for several months and gradually recovered [4]. Another study, which was conducted in 2022, described a 69-year-old man with fedratinib-induced orbital inflammation [3]. After discontinuing fedratinib therapy, the patient’s symptoms improved rapidly.

In our case report, we described the case of a 74-year-old woman with fedratinib-associated uveitis in both eyes. She was treated with STA injection on both eyes which led to the disappearance of the patient’s subjective symptoms the next day, and her objective symptoms improved from the seven-day follow-up onward.

## Conclusions

In this case, fedratinib therapy was continued because it effectively treated splenomegaly. All potential causes were comprehensively investigated and excluded from the diagnosis. When examining patients with uveitis undergoing fedratinib therapy for MF, physicians must consider the possibility that the cause of uveitis may be drug-induced. STA injection was an extremely effective treatment option for fedratinib-associated uveitis in the present case. 
